# Synthesis, crystal structure at 219 K and Hirshfeld surface analyses of 1,4,6-tri­methyl­quinoxaline-2,3(1*H*,4*H*)-dione monohydrate

**DOI:** 10.1107/S2056989020009573

**Published:** 2020-07-17

**Authors:** Ayman Zouitini, Md. Serajul Haque Faizi, Younes Ouzidan, Fouad Ouazzani Chahdi, Jérôme Marrot, Damien Prim, Necmi Dege, Ashraf Mashrai

**Affiliations:** a Laboratoire de Chimie Organique Appliquée, Université Sidi Mohamed Ben Abdallah, Faculté des Sciences et Techniques, BP 2202, Fez, Morocco; b Department of Chemistry, Langat Singh College, B.R.A. Bihar University, Muzaffarpur, Bihar-842001, India; c Laboratoire de Chimie Physique et Chimie Bio-organique, Faculté des Sciences et Techniques Mohammedia, Université Hassan II, Casablanca, BP 146, 28800, Mohammedia, Morocco; d Institut Lavoisier de Versailles, UVSQ, CNRS, Université Paris-Saclay, 78035 Versailles, France; e Department of Physics, Faculty of Arts and Sciences, Ondokuz Mayıs University, Samsun, 55200, Turkey; f Department of Pharmacy, University of Science and Technology, Ibb Branch, Ibb, Yemen

**Keywords:** crystal structure, quinoxaline-2,3-dione, Hirshfeld surface analysis, disorder, hydrogen bonding

## Abstract

The asymmetric unit of the title compound, C_11_H_12_N_2_O_2_·H_2_O, contains a mol­ecule of 1,4,6-trimethyl-1,4-di­hydro­quinoxaline-2,3-dione and a solvent water mol­ecule. Four atoms of the benzene ring are disordered over two sets of sites in a 0.706 (7):0.294 (7) ratio while the N-bound methyl groups are rotationally disordered with occupancy ratios of 0.78 (4):0.22 (4) and 0.76 (5):0.24 (5). In the crystal, mol­ecules are linked by O—H⋯O and C—H⋯O hydrogen bonds into layers lying parallel to (10). The Hirshfeld surface analysis indicates that the most important contributions to the packing arrangement are due to H⋯H (51.3%) and O⋯H/H⋯O (28.6%) inter­actions. The mol­ecular structure calculated by density functional theory is compared with the experimentally determined mol­ecular structure, and the HOMO–LUMO energy gap has been calculated.

## Abstract 2

The asymmetric unit of the title compound, C_11_H_12_N_2_O_2_·H_2_O, contains a disordered mol­ecule of 1,4,6-trimethyl-1,4-di­hydro-quinoxaline-2,3-dione and a solvent water mol­ecule. In the crystal, mol­ecules are linked by O—H⋯O and C—H⋯O hydrogen bonds into layers lying parallel to (10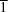). The Hirshfeld surface analysis is carried out.

## Chemical context   

Quinoxalines are well-known important nitro­gen-containing heterocyclic compounds with fused benzene and pyrazine rings. Quinoxalines and their derivatives display various pharmacological and biological activities, such as anti­cancer (Carta *et al.*, 2006 ▸), anti­diabetic (Bahekar *et al.*, 2007 ▸), anti­viral (Fonseca *et al.*, 2004 ▸), anti­bacterial (El-Sabbagh *et al.*, 2009 ▸), anti-inflammatory (Wagle *et al.*, 2008 ▸) and anti­protozoal (Hui *et al.*, 2006 ▸). The present work is a part of an ongoing structural study of quinoxaline derivatives (Faizi & Parashchenko 2015 ▸; Faizi *et al.*, 2015 ▸, 2018 ▸).
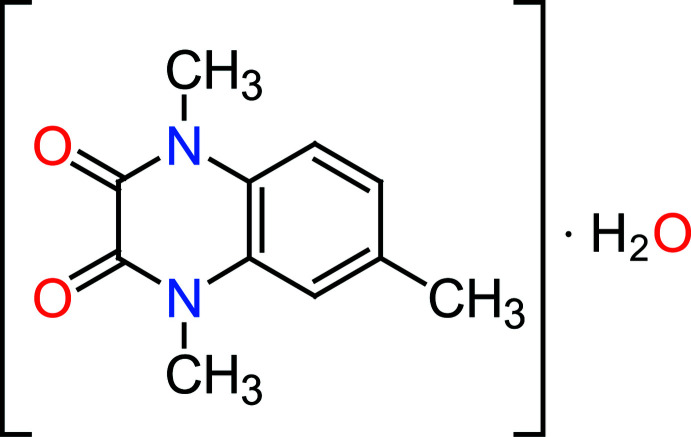


As a continuation of our research devoted to the synthesis and applications of new heterocyclic compounds obtained by *N*-alkyl­ation reactions (Tribak *et al.*, 2017 ▸; Qachchachi *et al.*, 2016 ▸; Belaziz *et al.*, 2012 ▸), we report here the synthesis of 1,4,6-tri­methyl­quinoxaline-2,3(1*H*,4*H*)-dione obtained by the action of iodo­methane on 6-methyl­quinoxaline-2,3(1*H*,4*H*)-dione, and the crystal structure of its monohydrate derivative along with the Hirshfeld surface analysis. The experimentally determined mol­ecular structure is compared with that calculated at the DFT/B3LYP/6-311 G(d,p) level.

## Structural commentary   

**Figure 1:**
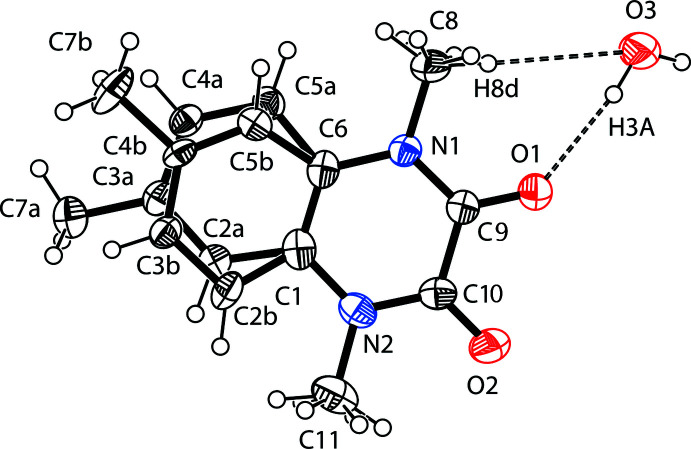
The asymmetric unit of the title compound, showing the atom labelling and displacement ellipsoids drawn at the 40% probability level. O—H⋯O hydrogen bonds are indicated by dashed lines. The benzene fragment of the organic mol­ecule, C2/C3/C4/C5/C7, is disordered over two sets of sites.

The title compound crystallizes in space group *P*2_1_/*n* with one quinoxaline and one water mol­ecule per asymmetric unit. The organic mol­ecule is disordered over two sets of sites with an occupancy ratio of 0.706 (7):0.294 (7). The disorder involves not only the orientation of the methyl group attached to the benzene ring, but also the positions of four carbon atoms of this ring, which are split (Fig. 1 ▸). Only the predominant orientation of the 1,4,6-tri­methyl­quinoxaline-2,3(1*H*,4*H*)-dione mol­ecule is discussed below. Besides this, the methyl groups attached to N1 and N2 nitro­gen atoms are also rotationally disordered with occupancy ratios of 0.78 (4):0.22 (4) and 0.76 (5):0.24 (5), respectively. The quinoxaline ring system is essentially planar, the largest deviation from the mean plane being 0.015 (3) Å for the N2 atom. The C=O and C*sp* ^2^—N bond lengths are typical of such type of compounds and indicate strong conjugation in the amide fragments.

## Supra­molecular features   

**Figure 2:**
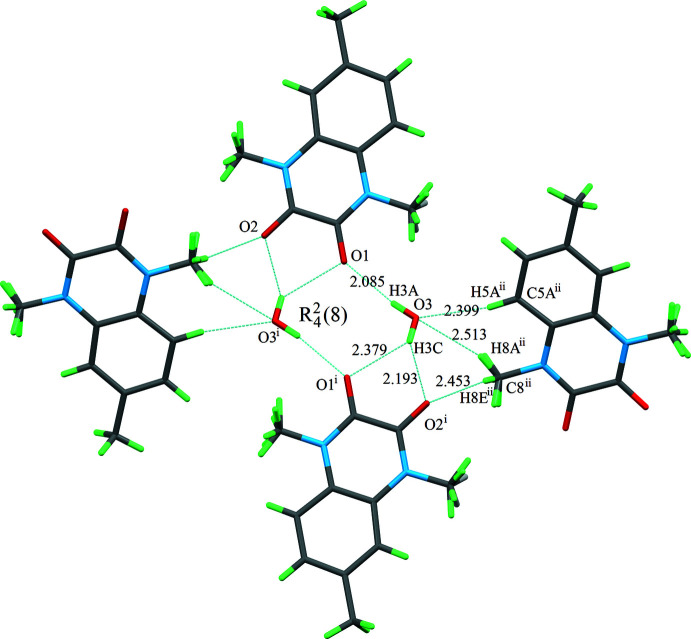
A view along the *a* axis of a hydrogen-bonded fragment. The O—H⋯O and C—H⋯O hydrogen bonds (shown as dashed lines) form an 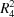
(8) ring motif.

**Figure 3:**
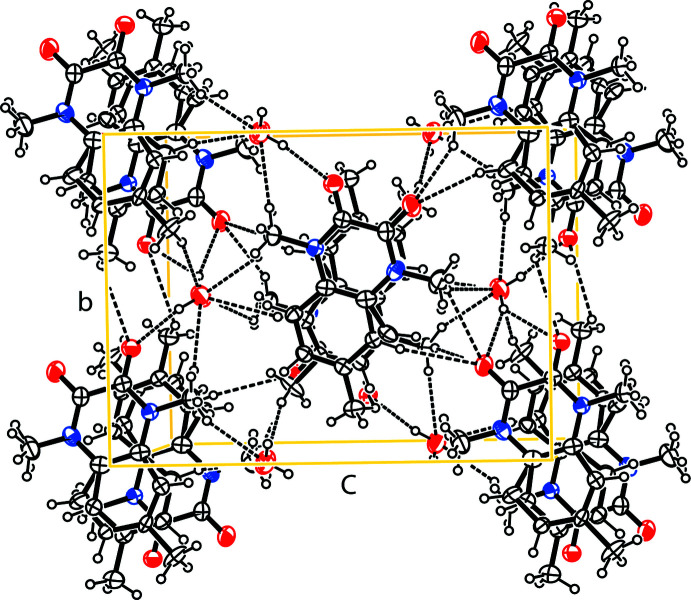
Packing diagram of the title compound viewed along the *a*-axis direction. Only the major disorder component is shown.

In the crystal, mol­ecules are linked by O—H⋯O and C—H⋯O hydrogen bonds (Table 1 ▸) into double layers lying parallel to (10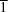). The smallest element of the hydrogen-bonding motif, where the 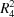
(8) rings are formed, is shown in Fig. 2 ▸, whereas the whole packing diagram is presented in Fig. 3 ▸. The water mol­ecule behaves both as a donor and an acceptor of hydrogen atoms in the hydrogen bonds. As seen in Fig. 3 ▸, in centrosymmetric pairs of organic mol­ecules, the aromatic and heterocyclic rings overlap with each other with an inter­centroid distance of 3.522 (4) Å, indicating that some π–π inter­actions occur.

## Hirshfeld surface analysis   

**Figure 4:**
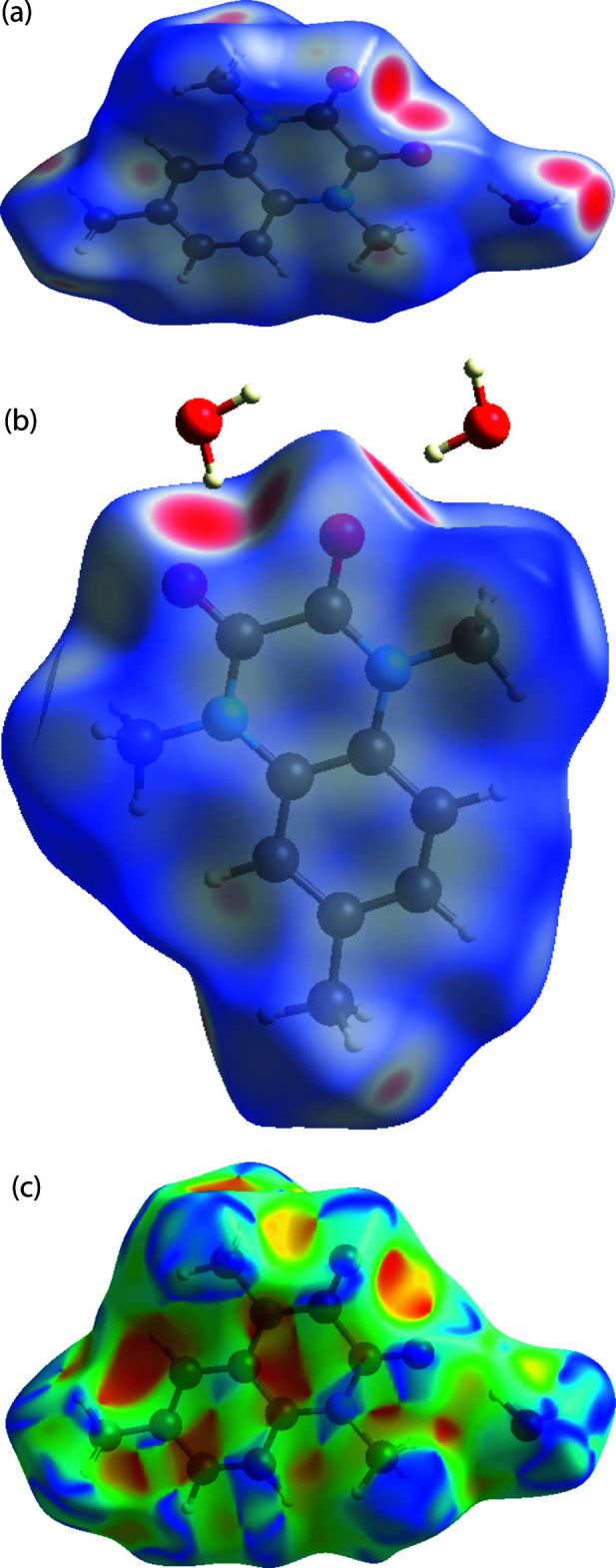
Views of the three-dimensional Hirshfeld surface for the title compound plotted over (*a*, *b*) *d*_norm_ and (*c*) shape-index.

The inter­molecular inter­actions were investigated qu­anti­tatively and visualized with *Crystal Explorer 17.5* (Turner *et al.*, 2017 ▸; Spackman *et al.*, 2009 ▸). The *d*_norm_, water inter­action, curvedness and 2D finger print plots are depicted in Fig. 4 ▸ *a*–*c* and 5 ▸ *a*–*h*, respectively. The red spots on the Hirshfeld surface represent O—H⋯O contacts while the blue regions correspond to weak inter­actions such as C—H⋯O contacts. The H⋯H inter­actions (51.3%) are the major factor in the crystal packing with O⋯H/H⋯O inter­actions (28.6%) representing the next highest contribution. The percentage contributions of other weak inter­actions are: C⋯C (8.2%), C⋯H/H⋯C (5.8%), C⋯N/N⋯C (4.5%), N⋯H/H⋯N (1.1%) and O⋯C/C⋯O (0.5%).

## DFT calculations   

**Figure 6:**
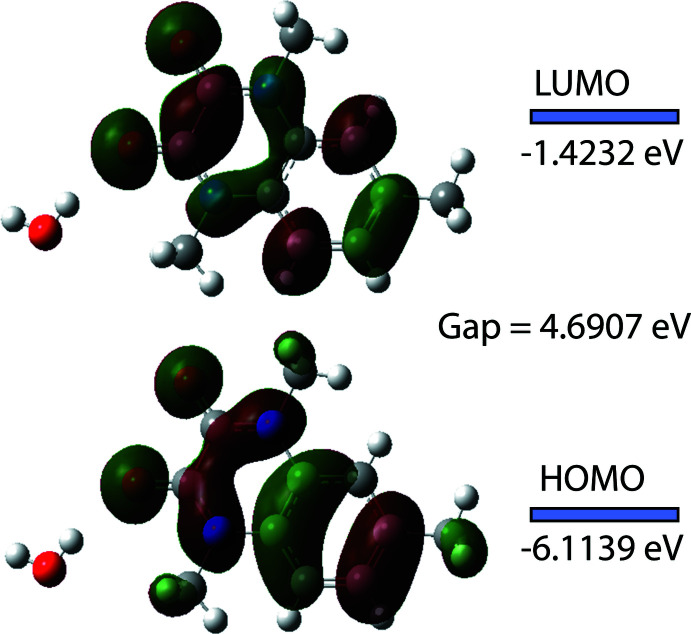
Frontier mol­ecular orbitals of the 1,4,6-tri­methyl­quinoxaline-2,3(1*H*,4*H*)-dione mol­ecule.

The structure of the title organic mol­ecule was optimized in the gas-phase approximation at the level of density functional theory (DFT) using the B3LYP functional (Becke, 1993 ▸) and 6-311 G(d,p) basis set as implemented in *GAUSSIAN 09* (Frisch *et al.*, 2009 ▸). The theoretical and experimental bond lengths and angles are in good agreement (Table 2 ▸). The energetic and spatial characteristics of the highest occupied mol­ecular orbital (HOMO), acting as an electron donor, and the lowest unoccupied mol­ecular orbital (LUMO), acting as an electron acceptor, are very important parameters for quantum chemistry. When the energy gap is small, the mol­ecule is highly polarizable and has high chemical reactivity (Fukui, 1982 ▸; Khan *et al.*, 2015 ▸). The DFT calculations provide some important information on the reactivity and site selectivity of the mol­ecular framework, *E*_HOMO_ and *E*_LUMO_, electronegativity (χ), hardness (η), electrophilicity (ω), softness (*σ*) and fraction of electrons transferred (*ΔN*). These data are given in Table 3 ▸. The parameters η and *σ* are significant for evaluation of both the reactivity and stability. The electron transition from HOMO to LUMO is shown in Fig. 6 ▸. The HOMO and LUMO are localized in the plane of the whole 1,4,6-tri­methyl­quinoxaline-2,3(1*H*,4*H*)-dione bicyclic ring system. The energy gap [Δ*E* = *E*_LUMO_ − *E*_HOMO_] of the mol­ecule is 4.6907 eV, the frontier mol­ecular orbital energies *E*_HOMO_ and *E*_LUMO_ being −6.1139 eV and −1.4232 eV, respectively. The dipole moment of (I) is estimated to be 5.56 Debye.

## Database survey   

A search of the Cambridge Structural Database (CSD, version 5.39; Groom *et al.*, 2016 ▸) gave nine hits for the 1,4,6-tri­methyl­quinoxaline-2,3(1*H*,4*H*)-dione moiety. Two of them are metal complexes, bis­(μ_2_-nitrato-*O*,*O*,*O*′)-bis­[1,4-bis­(*N*,*N*- diisopropyl-acetamido)­quinoxaline-2,3-dione-*O*,*O*′]tetra­kis(nitrato-*O*,*O*′)di­aqua­dineodymium(III) monohydrate (WIKSOZ; Song *et al.*, 2007 ▸) and *catena*-(μ_2_-iodo)-bis­(1,4-di­methyl­quinoxalin-2,3-dionato)potassium (FADQOS; Benali *et al.*, 2008 ▸). Seven organic compounds similar to the title compound are reported in the literature. In 1,4-dihexyl-1,4-di­hydro­quinoxaline-2,3-dione (FECROX; El Bourakadi *et al.*, 2017*a* ▸), the methyl groups attached to the N atoms are replaced by hexyl groups. In 1,4-di­allyl­quinoxaline-2,3(1*H*,4*H*)-dione (GURGAB; Mustaphi *et al.*, 2001 ▸), the allyl groups are bound to the N atoms. In 1-ethyl-4-phenyl­ethyl-1,4-di­hydro­quinoxaline-2,3-dione (IXATOQ; Akkurt *et al.*, 2004 ▸), one N atom is bound to an ethyl group, and the other to an ethyl­phenyl group. In 6-methyl-1,4-bis­[(pyridin-2-yl)meth­yl]-1,4-di­hydro­quinoxaline-2,3-dione (KELMIA; Zouitini *et al.*, 2017 ▸), methyl­pyridinyl groups are attached to both N atoms. In 1,4-dibenzyl-6-chloro-1,4-di­hydro­quinoxaline-2,3-dione (PAWFEB; El Janati *et al.*, 2017*a* ▸), the N atoms are attached to the benzyl groups, and the methyl group on the benzene ring is substituted by chlorine. In 1,4-dioctyl-1,4-di­hydro­quinoxaline-2,3-dione (WAPWAO; El Bourakadi *et al.*, 2017*b* ▸), octyl groups are attached to the N atoms. In 6-chloro-1,4-diethyl-1,4-di­hydro­quinoxaline-2,3-dione (XEFMON; El Janati *et al.*, 2017*b* ▸), the ethyl groups are attached to the N atoms, and the methyl group on the benzene ring is substituted by chlorine, as in PAWFEB. None of these structures contains solvent mol­ecules.

## Synthesis and crystallization   

To a solution of 6-methyl-1,4-di­hydro­quinoxaline-2,3-dione (0.3 g, 1.73 mmol) in DMF (15 ml) potassium carbonate (0.47 g, 3.61 mmol) and tetra-*n*-butyl­ammonium (0.07g, 0.23 mmol) were added. After 10 min of stirring, 0.27 ml (4.32 mmol) of iodo­methane were added, and the mixture was stirred at room temperature for 6 h. The inorganic salts were filtered off, DMF was evaporated under reduced pressure and the residue was dissolved in di­chloro­methane. The organic phase was dried over Na_2_SO_4_ and then concentrated. The crude product was purified by chromatography on a silica gel column [eluent: hexa­ne/ethyl­acetate (2/1)].

## Refinement   

Crystal data, data collection and structure refinement details are summarized in Table 4 ▸. Water mol­ecules were refined as rigid groups with *U*_iso_(H) = 1.5*U*_eq_(O). Other H atoms were positioned geometrically, with C—H = 0.94 and 0.97 Å for aromatic and aliphatic H atoms, respectively, and constrained to ride on their parent atoms, with *U*_iso_(H) = 1.2*U*_eq_(C) or *U*_iso_(H) = 1.5*U*_eq_(C-meth­yl). The disorder of the organic mol­ecule was taken into account using free variables.

## Supplementary Material

Crystal structure: contains datablock(s) I. DOI: 10.1107/S2056989020009573/yk2133sup1.cif

Structure factors: contains datablock(s) I. DOI: 10.1107/S2056989020009573/yk2133Isup2.hkl

CCDC reference: 1936663

Additional supporting information:  crystallographic information; 3D view; checkCIF report

## Figures and Tables

**Figure 5:**
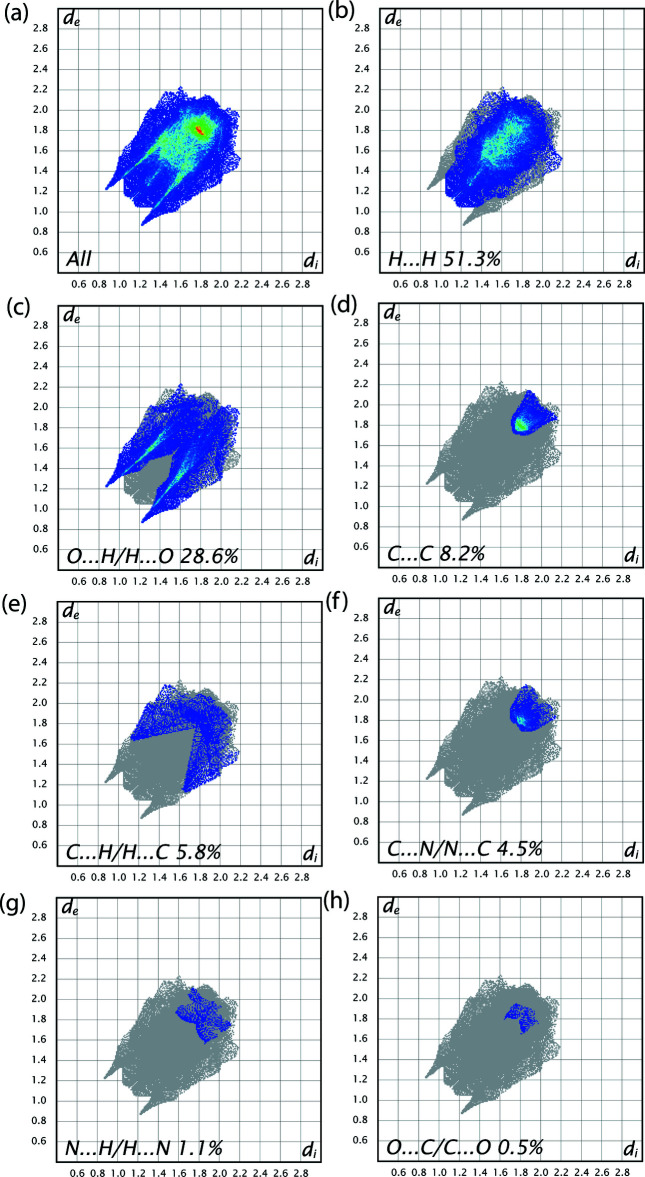
Two-dimensional fingerprint plots showing (*a*) all inter­actions and those delineated into (*b*) H⋯H, (*c*) O⋯H/H⋯O, (*d*) C⋯C, (*e*) C⋯H/H⋯C, (*f*) C⋯N/N⋯C,(*g*) N⋯H/H⋯N and (*h*) O⋯C/C⋯O.

**Table 1:** Hydrogen-bond geometry (Å, °)

*D*—H⋯*A*	*D*—H	H⋯*A*	*D*⋯*A*	*D*—H⋯*A*
O3—H3*A*⋯O1	0.86	2.09	2.936 (4)	170
O3—H3*C*⋯O1^i^	0.86	2.38	3.062 (4)	137
O3—H3*C*⋯O2^i^	0.86	2.19	2.972 (5)	150
C5*A*—H5*A*⋯O3^ii^	0.94	2.40	3.298 (13)	160
C8—H8*E*⋯O2^iii^	0.97	2.45	3.335 (4)	151

Symmetry codes: (i) 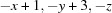; (ii) 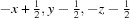; (iii) 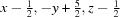.

**Table 2:** Comparison of observed (X-ray data) and calculated (DFT) geometric parameters (Å, °)

Parameter	X-ray	B3LYP/6–311G(d,p)
O1—C9	1.228 (4)	1.217
O2—C10	1.226 (4)	1.211
N1—C6	1.401 (4)	1.407
N1—C8	1.470 (4)	1.468
N1—C9	1.351 (4)	1.375
N2—C1	1.409 (4)	1.375
N2—C10	1.365 (5)	1.384
N2—C11	1.458 (5)	1.464
O1—C9—N1	123.5 (3)	123.9
O2—C10—N2	122.8 (3)	123.4
O1—C9—C10	118.3 (3)	118.3

**Table 3:** DFT-calculated mol­ecular characteristics for the title compound

Total Energy, *TE* (eV)	−20757.4747
*E*_HOMO_ (eV)	−6.1139
*E*_LUMO_ (eV)	−1.4232
Gap, *ΔE* (eV)	4.6907
Dipole moment, *μ* (D)	5.56
Ionization potential, *I* (eV)	6.1139
Electron affinity, *A* (eV)	1.4232
Electronegativity, *χ*	3.929
Hardness, *η*	2.345
Electrophilicity index, *ω*	3.291
Softness, *σ*	0.213
Fraction of electron transferred, *ΔN*	0.655

**Table 4:** Experimental details

Crystal data	
Chemical formula	C_11_H_12_N_2_O_2_·H_2_O
*M*_r_	222.24
Crystal system, space group	Monoclinic, *P*2_1_/*n*
Temperature (K)	219
*a*, *b*, *c* (Å)	7.0695 (4), 10.8321 (5), 14.4349 (6)
β (°)	101.556 (3)
*V* (Å^3^)	1082.98 (9)
*Z*	4
Radiation type	Mo *K*α
μ (mm^−1^)	0.10
Crystal size (mm)	0.30 × 0.18 × 0.04

Data collection	
Diffractometer	Bruker APEXII CCD
Absorption correction	Multi-scan (*SADABS*; Krause *et al.*, 2015 ▸)
No. of measured, independent and observed [*I* > 2σ(*I*)] reflections	32798, 1935, 1563
*R*_int_	0.062
(sin θ/λ)_max_ (Å^−1^)	0.598

Refinement	
*R*[*F* ^2^ > 2σ(*F* ^2^)], *wR*(*F* ^2^), *S*	0.067, 0.147, 1.24
No. of reflections	1935
No. of parameters	201
No. of restraints	30
H-atom treatment	H-atom parameters constrained
Δρ_max_, Δρ_min_ (e Å^−3^)	0.21, −0.18

Computer programs: *APEX3* and *SAINT* (Bruker, 2016 ▸), *SHELXT* (Sheldrick, 2015*a* ▸), *SHELXL2018* (Sheldrick, 2015*b* ▸), *OLEX2* (Dolomanov *et al.*, 2009 ▸), *Mercury* (Macrae *et al.*, 2020 ▸), *WinGX* (Farrugia, 2012 ▸), *PLATON* (Spek, 2020 ▸) and *publCIF* (Westrip, 2010 ▸).

## supplementary crystallographic information

### Crystal data

**Table:**

C_11_H_12_N_2_O_2_·H_2_O	*F*(000) = 472
*M**_r_* = 222.24	*D*_x_ = 1.363 Mg m^−^^3^
Monoclinic, *P*2_1_/*n*	Mo *K*α radiation, λ = 0.71073 Å
*a* = 7.0695 (4) Å	Cell parameters from 7373 reflections
*b* = 10.8321 (5) Å	θ = 2.4–24.7°
*c* = 14.4349 (6) Å	µ = 0.10 mm^−^^1^
β = 101.556 (3)°	*T* = 219 K
*V* = 1082.98 (9) Å^3^	Parallelepiped, yellow
*Z* = 4	0.30 × 0.18 × 0.04 mm

### Data collection

**Table:**

Bruker APEXII CCD diffractometer	1563 reflections with *I* > 2σ(*I*)
Radiation source: fine-focus sealed tube	*R*_int_ = 0.062
φ and ω scans	θ_max_ = 25.2°, θ_min_ = 2.4°
Absorption correction: multi-scan (SADABS; Krause *et al.*, 2015)	*h* = −8→8
	*k* = −12→12
32798 measured reflections	*l* = −17→17
1935 independent reflections	

### Refinement

**Table:**

Refinement on *F*^2^	Secondary atom site location: difference Fourier map
Least-squares matrix: full	Hydrogen site location: mixed
*R*[*F*^2^ > 2σ(*F*^2^)] = 0.067	H-atom parameters constrained
*wR*(*F*^2^) = 0.147	*w* = 1/[σ^2^(*F*_o_^2^) + (0.0097*P*)^2^ + 1.8308*P*] where *P* = (*F*_o_^2^ + 2*F*_c_^2^)/3
*S* = 1.24	(Δ/σ)_max_ < 0.001
1935 reflections	Δρ_max_ = 0.21 e Å^−^^3^
201 parameters	Δρ_min_ = −0.18 e Å^−^^3^
30 restraints	Extinction correction: SHELXL2018 (Sheldrick, 2015b), Fc^*^=kFc[1+0.001xFc^2^λ^3^/sin(2θ)]^-1/4^
Primary atom site location: structure-invariant direct methods	Extinction coefficient: 0.0031 (9)

### Special details

**Table:**

Geometry. All esds (except the esd in the dihedral angle between two l.s. planes) are estimated using the full covariance matrix. The cell esds are taken into account individually in the estimation of esds in distances, angles and torsion angles; correlations between esds in cell parameters are only used when they are defined by crystal symmetry. An approximate (isotropic) treatment of cell esds is used for estimating esds involving l.s. planes.

### Fractional atomic coordinates and isotropic or equivalent isotropic displacement parameters (Å^2^)

**Table:**

	*x*	*y*	*z*	*U*_iso_*/*U*_eq_	Occ. (<1)
O1	0.3712 (4)	1.3409 (2)	0.00205 (17)	0.0479 (7)	
O2	0.3853 (4)	1.2710 (3)	0.18106 (17)	0.0581 (8)	
C1	0.2612 (4)	0.9861 (3)	0.0618 (3)	0.0409 (8)	
C2A	0.2177 (14)	0.8598 (10)	0.0725 (6)	0.034 (2)	0.706 (7)
H2A	0.219201	0.830335	0.133895	0.041*	0.706 (7)
C2B	0.228 (5)	0.879 (3)	0.1074 (16)	0.048 (7)	0.294 (7)
H2B	0.237848	0.867991	0.172811	0.057*	0.294 (7)
C3A	0.1723 (13)	0.7756 (12)	−0.0031 (7)	0.037 (2)	0.706 (7)
C3B	0.175 (4)	0.788 (3)	0.0367 (16)	0.037 (5)	0.294 (7)
H3B	0.152151	0.706837	0.055446	0.045*	0.294 (7)
C4A	0.1703 (14)	0.8174 (10)	−0.0933 (6)	0.038 (2)	0.706 (7)
H4A	0.142000	0.761812	−0.144148	0.045*	0.706 (7)
C4B	0.156 (3)	0.811 (2)	−0.0554 (16)	0.035 (6)	0.294 (7)
C5A	0.210 (2)	0.9418 (16)	−0.1111 (9)	0.039 (3)	0.706 (7)
H5A	0.207157	0.970180	−0.172834	0.047*	0.706 (7)
C5B	0.193 (5)	0.923 (4)	−0.087 (2)	0.032 (6)	0.294 (7)
H5B	0.176018	0.933494	−0.153049	0.038*	0.294 (7)
C6	0.2535 (4)	1.0233 (3)	−0.0314 (2)	0.0369 (8)	
N1	0.2915 (4)	1.1465 (2)	−0.05089 (18)	0.0359 (7)	
C9	0.3382 (4)	1.2324 (3)	0.0177 (2)	0.0354 (7)	
C10	0.3472 (5)	1.1936 (3)	0.1182 (2)	0.0402 (8)	
N2	0.3101 (4)	1.0729 (3)	0.1354 (2)	0.0440 (7)	
C8	0.2761 (6)	1.1869 (4)	−0.1493 (2)	0.0540 (10)	
H8A	0.223018	1.120497	−0.191729	0.081*	0.22 (4)
H8B	0.403194	1.208575	−0.159941	0.081*	0.22 (4)
H8C	0.191973	1.258317	−0.161254	0.081*	0.22 (4)
H8D	0.322439	1.271095	−0.150220	0.081*	0.78 (4)
H8E	0.142263	1.183017	−0.182009	0.081*	0.78 (4)
H8F	0.353484	1.133275	−0.180695	0.081*	0.78 (4)
C11	0.3169 (7)	1.0381 (4)	0.2335 (3)	0.0696 (13)	
H11A	0.279949	0.952205	0.236449	0.104*	0.24 (5)
H11B	0.228285	1.089532	0.259797	0.104*	0.24 (5)
H11C	0.446948	1.049549	0.269709	0.104*	0.24 (5)
H11D	0.356839	1.108652	0.274187	0.104*	0.76 (5)
H11E	0.408503	0.971326	0.250839	0.104*	0.76 (5)
H11F	0.189839	1.011308	0.240928	0.104*	0.76 (5)
O3	0.3982 (6)	1.4989 (3)	−0.1600 (2)	0.0833 (11)	
H3A	0.382975	1.460410	−0.109907	0.125*	
H3C	0.477462	1.559146	−0.144450	0.125*	
C7A	0.1315 (8)	0.6428 (5)	0.0165 (4)	0.0528 (17)	0.706 (7)
H7A2	0.035271	0.639039	0.055782	0.079*	0.706 (7)
H7A3	0.249382	0.603381	0.049019	0.079*	0.706 (7)
H7A1	0.083495	0.600399	−0.042761	0.079*	0.706 (7)
C7B	0.0926 (19)	0.7135 (13)	−0.1317 (10)	0.062 (5)	0.294 (7)
H7B1	0.025634	0.753011	−0.189272	0.093*	0.294 (7)
H7B2	0.006626	0.655227	−0.110056	0.093*	0.294 (7)
H7B3	0.205065	0.670198	−0.143967	0.093*	0.294 (7)

### Atomic displacement parameters (Å^2^)

**Table:**

	*U*^11^	*U*^22^	*U*^33^	*U*^12^	*U*^13^	*U*^23^
O1	0.0528 (15)	0.0419 (15)	0.0475 (14)	−0.0027 (12)	0.0067 (11)	0.0004 (12)
O2	0.0675 (18)	0.0632 (18)	0.0414 (14)	−0.0097 (14)	0.0057 (12)	−0.0075 (14)
C1	0.0219 (15)	0.0408 (19)	0.059 (2)	0.0014 (14)	0.0050 (14)	0.0022 (17)
C2A	0.026 (3)	0.038 (6)	0.038 (5)	0.003 (3)	0.007 (4)	0.000 (5)
C2B	0.051 (9)	0.040 (10)	0.051 (13)	−0.009 (7)	0.008 (11)	−0.016 (11)
C3A	0.029 (3)	0.039 (5)	0.043 (8)	−0.003 (3)	0.006 (5)	−0.005 (6)
C3B	0.040 (8)	0.034 (10)	0.036 (13)	0.003 (7)	0.003 (10)	−0.004 (11)
C4A	0.035 (3)	0.039 (4)	0.037 (5)	0.003 (2)	0.002 (4)	−0.005 (4)
C4B	0.033 (7)	0.042 (11)	0.029 (14)	−0.002 (7)	0.008 (10)	−0.013 (11)
C5A	0.038 (5)	0.038 (5)	0.039 (6)	−0.002 (4)	0.002 (4)	0.006 (4)
C5B	0.014 (7)	0.042 (14)	0.038 (13)	0.005 (7)	0.003 (8)	0.005 (10)
C6	0.0200 (15)	0.0367 (18)	0.050 (2)	0.0025 (13)	−0.0020 (13)	−0.0011 (15)
N1	0.0297 (14)	0.0393 (15)	0.0358 (15)	0.0005 (12)	−0.0002 (11)	−0.0006 (12)
C9	0.0259 (16)	0.0385 (19)	0.0407 (18)	0.0022 (14)	0.0037 (13)	−0.0001 (15)
C10	0.0318 (17)	0.052 (2)	0.0362 (18)	−0.0002 (15)	0.0048 (14)	−0.0006 (16)
N2	0.0360 (15)	0.0523 (18)	0.0448 (17)	0.0004 (14)	0.0108 (13)	0.0091 (14)
C8	0.063 (3)	0.057 (2)	0.037 (2)	0.002 (2)	−0.0016 (17)	0.0010 (17)
C11	0.081 (3)	0.078 (3)	0.054 (3)	0.002 (3)	0.023 (2)	0.017 (2)
O3	0.127 (3)	0.069 (2)	0.0480 (17)	−0.041 (2)	0.0053 (18)	0.0030 (16)
C7A	0.055 (3)	0.041 (3)	0.063 (4)	−0.001 (2)	0.013 (3)	0.002 (3)
C7B	0.048 (8)	0.063 (9)	0.077 (10)	−0.005 (7)	0.014 (7)	−0.045 (8)

### Geometric parameters (Å, º)

**Table:**

O1—C9	1.228 (4)	N1—C8	1.470 (4)
O2—C10	1.226 (4)	C9—C10	1.499 (5)
C1—C2B	1.38 (3)	C10—N2	1.365 (5)
C1—C6	1.394 (5)	N2—C11	1.458 (5)
C1—N2	1.409 (4)	C8—H8A	0.9700
C1—C2A	1.418 (12)	C8—H8B	0.9700
C2A—C3A	1.409 (11)	C8—H8C	0.9700
C2A—H2A	0.9400	C8—H8D	0.9700
C2B—C3B	1.41 (2)	C8—H8E	0.9700
C2B—H2B	0.9400	C8—H8F	0.9700
C3A—C4A	1.375 (11)	C11—H11A	0.9700
C3A—C7A	1.504 (14)	C11—H11B	0.9700
C3B—C4B	1.34 (3)	C11—H11C	0.9700
C3B—H3B	0.9400	C11—H11D	0.9700
C4A—C5A	1.41 (2)	C11—H11E	0.9700
C4A—H4A	0.9400	C11—H11F	0.9700
C4B—C5B	1.34 (4)	O3—H3A	0.8603
C4B—C7B	1.53 (2)	O3—H3C	0.8598
C5A—C6	1.434 (17)	C7A—H7A2	0.9700
C5A—H5A	0.9400	C7A—H7A3	0.9700
C5B—C6	1.37 (4)	C7A—H7A1	0.9700
C5B—H5B	0.9400	C7B—H7B1	0.9700
C6—N1	1.401 (4)	C7B—H7B2	0.9700
N1—C9	1.351 (4)	C7B—H7B3	0.9700
			
C2B—C1—C6	136.5 (10)	H8B—C8—H8C	109.5
C2B—C1—N2	104.1 (10)	N1—C8—H8D	109.5
C6—C1—N2	119.4 (3)	H8A—C8—H8D	141.1
C6—C1—C2A	114.6 (5)	H8B—C8—H8D	56.3
N2—C1—C2A	126.0 (5)	H8C—C8—H8D	56.3
C3A—C2A—C1	124.1 (8)	N1—C8—H8E	109.5
C3A—C2A—H2A	117.9	H8A—C8—H8E	56.3
C1—C2A—H2A	117.9	H8B—C8—H8E	141.1
C1—C2B—C3B	107 (2)	H8C—C8—H8E	56.3
C1—C2B—H2B	126.6	H8D—C8—H8E	109.5
C3B—C2B—H2B	126.6	N1—C8—H8F	109.5
C4A—C3A—C2A	118.5 (11)	H8A—C8—H8F	56.3
C4A—C3A—C7A	121.8 (10)	H8B—C8—H8F	56.3
C2A—C3A—C7A	119.7 (9)	H8C—C8—H8F	141.1
C4B—C3B—C2B	123 (3)	H8D—C8—H8F	109.5
C4B—C3B—H3B	118.5	H8E—C8—H8F	109.5
C2B—C3B—H3B	118.5	N2—C11—H11A	109.5
C3A—C4A—C5A	121.5 (11)	N2—C11—H11B	109.5
C3A—C4A—H4A	119.2	H11A—C11—H11B	109.5
C5A—C4A—H4A	119.2	N2—C11—H11C	109.5
C3B—C4B—C5B	122 (3)	H11A—C11—H11C	109.5
C3B—C4B—C7B	123 (2)	H11B—C11—H11C	109.5
C5B—C4B—C7B	115 (2)	N2—C11—H11D	109.5
C4A—C5A—C6	117.4 (9)	H11A—C11—H11D	141.1
C4A—C5A—H5A	121.3	H11B—C11—H11D	56.3
C6—C5A—H5A	121.3	H11C—C11—H11D	56.3
C4B—C5B—C6	125 (2)	N2—C11—H11E	109.5
C4B—C5B—H5B	117.5	H11A—C11—H11E	56.3
C6—C5B—H5B	117.5	H11B—C11—H11E	141.1
C5B—C6—C1	106.7 (11)	H11C—C11—H11E	56.3
C5B—C6—N1	133.4 (11)	H11D—C11—H11E	109.5
C1—C6—N1	119.8 (3)	N2—C11—H11F	109.5
C1—C6—C5A	123.8 (6)	H11A—C11—H11F	56.3
N1—C6—C5A	116.4 (6)	H11B—C11—H11F	56.3
C9—N1—C6	122.5 (3)	H11C—C11—H11F	141.1
C9—N1—C8	117.6 (3)	H11D—C11—H11F	109.5
C6—N1—C8	119.9 (3)	H11E—C11—H11F	109.5
O1—C9—N1	123.5 (3)	H3A—O3—H3C	109.5
O1—C9—C10	118.3 (3)	C3A—C7A—H7A2	109.5
N1—C9—C10	118.2 (3)	C3A—C7A—H7A3	109.5
O2—C10—N2	122.8 (3)	H7A2—C7A—H7A3	109.5
O2—C10—C9	119.0 (3)	C3A—C7A—H7A1	109.5
N2—C10—C9	118.2 (3)	H7A2—C7A—H7A1	109.5
C10—N2—C1	121.9 (3)	H7A3—C7A—H7A1	109.5
C10—N2—C11	117.0 (3)	C4B—C7B—H7B1	109.5
C1—N2—C11	121.0 (3)	C4B—C7B—H7B2	109.5
N1—C8—H8A	109.5	H7B1—C7B—H7B2	109.5
N1—C8—H8B	109.5	C4B—C7B—H7B3	109.5
H8A—C8—H8B	109.5	H7B1—C7B—H7B3	109.5
N1—C8—H8C	109.5	H7B2—C7B—H7B3	109.5
H8A—C8—H8C	109.5		
			
C6—C1—C2A—C3A	0.8 (11)	C5B—C6—N1—C9	175.2 (19)
N2—C1—C2A—C3A	−179.0 (7)	C1—C6—N1—C9	0.7 (4)
C6—C1—C2B—C3B	1 (4)	C5A—C6—N1—C9	−178.8 (8)
N2—C1—C2B—C3B	179.2 (19)	C5B—C6—N1—C8	−3 (2)
C1—C2A—C3A—C4A	0.2 (15)	C1—C6—N1—C8	−177.6 (3)
C1—C2A—C3A—C7A	179.0 (7)	C5A—C6—N1—C8	2.8 (9)
C1—C2B—C3B—C4B	−3 (4)	C6—N1—C9—O1	−179.7 (3)
C2A—C3A—C4A—C5A	−0.9 (17)	C8—N1—C9—O1	−1.3 (5)
C7A—C3A—C4A—C5A	−179.7 (10)	C6—N1—C9—C10	−0.6 (4)
C2B—C3B—C4B—C5B	2 (5)	C8—N1—C9—C10	177.8 (3)
C2B—C3B—C4B—C7B	−178 (2)	O1—C9—C10—O2	0.6 (5)
C3A—C4A—C5A—C6	0.5 (19)	N1—C9—C10—O2	−178.5 (3)
C3B—C4B—C5B—C6	0 (5)	O1—C9—C10—N2	179.9 (3)
C7B—C4B—C5B—C6	−179 (2)	N1—C9—C10—N2	0.8 (4)
C4B—C5B—C6—C1	−2 (3)	O2—C10—N2—C1	178.2 (3)
C4B—C5B—C6—N1	−177.0 (19)	C9—C10—N2—C1	−1.0 (4)
C2B—C1—C6—C5B	1 (2)	O2—C10—N2—C11	0.1 (5)
N2—C1—C6—C5B	−176.8 (15)	C9—C10—N2—C11	−179.1 (3)
C2B—C1—C6—N1	177.2 (19)	C2B—C1—N2—C10	−177.6 (14)
N2—C1—C6—N1	−0.9 (4)	C6—C1—N2—C10	1.1 (4)
C2A—C1—C6—N1	179.2 (5)	C2A—C1—N2—C10	−179.0 (5)
N2—C1—C6—C5A	178.5 (9)	C2B—C1—N2—C11	0.4 (14)
C2A—C1—C6—C5A	−1.3 (10)	C6—C1—N2—C11	179.1 (3)
C4A—C5A—C6—C1	0.7 (17)	C2A—C1—N2—C11	−1.1 (7)
C4A—C5A—C6—N1	−179.8 (9)		

### Hydrogen-bond geometry (Å, º)

**Table:**

*D*—H···*A*	*D*—H	H···*A*	*D*···*A*	*D*—H···*A*
O3—H3*A*···O1	0.86	2.09	2.936 (4)	170
O3—H3*C*···O1^i^	0.86	2.38	3.062 (4)	137
O3—H3*C*···O2^i^	0.86	2.19	2.972 (5)	150
C5*A*—H5*A*···O3^ii^	0.94	2.40	3.298 (13)	160
C8—H8*E*···O2^iii^	0.97	2.45	3.335 (4)	151

Symmetry codes: (i) −*x*+1, −*y*+3, −*z*; (ii) −*x*+1/2, *y*−1/2, −*z*−1/2; (iii) *x*−1/2, −*y*+5/2, *z*−1/2.
